# Accuracy of genome-wide imputation in Braford and Hereford beef cattle

**DOI:** 10.1186/s12863-014-0157-9

**Published:** 2014-12-29

**Authors:** Mario L Piccoli, José Braccini, Fernando F Cardoso, Medhi Sargolzaei, Steven G Larmer, Flávio S Schenkel

**Affiliations:** Departamento de Zootecnia, Universidade Federal do Rio Grande do Sul, Porto Alegre, Brazil; GenSys Consultores Associados S/S, Porto Alegre, Brazil; Centre for Genetic Improvement of Livestock, University of Guelph, Guelph, ON Canada; Embrapa Southern Region Animal Husbandry, Bagé, Brazil; National Council for Scientific and Technological Development, Brasília, Brazil; The Semex Alliance, Guelph, ON Canada

**Keywords:** Braford, Imputation accuracy, Low density panel, Hereford, High density panel

## Abstract

**Background:**

Strategies for imputing genotypes from the Illumina-Bovine3K, Illumina-BovineLD (6K), BeefLD-GGP (8K), a non-commercial-15K and IndicusLD-GGP (20K) to either Illumina-BovineSNP50 (50K) or to Illumina-BovineHD (777K) SNP panel, as well as for imputing from 50K, GGP-IndicusHD (90iK) and GGP-BeefHD (90tK) to 777K were investigated. Imputation of low density (<50K) genotypes to 777K was carried out in either one or two steps. Imputation of ungenotyped parents (n = 37 sires) with four or more offspring to the 50K panel was also assessed. There were 2,946 Braford, 664 Hereford and 88 Nellore animals, from which 71, 59 and 88 were genotyped with the 777K panel, while all others had 50K genotypes. The reference population was comprised of 2,735 animals and 175 bulls for 50K and 777K, respectively. The low density panels were simulated by masking genotypes in the 50K or 777K panel for animals born in 2011. Analyses were performed using both Beagle and FImpute software. Genotype imputation accuracy was measured by concordance rate and allelic R^2^ between true and imputed genotypes.

**Results:**

The average concordance rate using FImpute was 0.943 and 0.921 averaged across all simulated low density panels to 50K or to 777K, respectively, in comparison with 0.927 and 0.895 using Beagle. The allelic R^2^ was 0.912 and 0.866 for imputation to 50K or to 777K using FImpute, respectively, and 0.890 and 0.826 using Beagle. One and two steps imputation to 777K produced averaged concordance rates of 0.806 and 0.892 and allelic R^2^ of 0.674 and 0.819, respectively. Imputation of low density panels to 50K, with the exception of 3K, had overall concordance rates greater than 0.940 and allelic R^2^ greater than 0.919. Ungenotyped animals were imputed to 50K panel with an average concordance rate of 0.950 by FImpute.

**Conclusion:**

FImpute accuracy outperformed Beagle on both imputation to 50K and to 777K. Two-step outperformed one-step imputation for imputing to 777K. Ungenotyped animals that have four or more offspring can have their 50K genotypes accurately inferred using FImpute. All low density panels, except the 3K, can be used to impute to the 50K using FImpute or Beagle with high concordance rate and allelic R^2^.

## Background

Traditional animal breeding methods utilized phenotypic data and relationships among individuals to make informed mating decision to improve traits of economic significance. Recent advances in DNA technology, led to the full sequencing of several species, including cattle [1] and to the development of new genomic technologies. SNP genotyping is now possible at a cost reasonable for producers. This includes the Illumina BovineHD (Illumina Inc., San Diego, USA), that makes it possible to genotype 777,962 SNPs in a single chip. The first panel of medium density for bovine was the Parallel 10K SNP released in 2006 by the Parallel Company. In 2007, the Illumina Inc., San Diego, USA developed the Illumina BovineSNP50 panel with 54,609 SNPs and in 2011 it released the Illumina BovineHD panel with 777,962 SNPs. These new genotyping technologies have stimulated the development of new research areas, including techniques to infer SNPs on high density genotype panels for animals that have been genotyped at a lower density.

Procedures for imputation of genotypes, a technique that refers to prediction of ungenotyped SNP genotypes, have been the subject of recent studies in some species, such as, dairy cattle [2,3], beef cattle [4,5], horse [6] and pig [7]. Software programs have been developed to more efficiently and accurately impute high density genotypes [8-12]. Density of markers genotyped affects genomic selection accuracy [13-15], and to reduce the cost of genotyping large populations, less dense, less expensive panels can be used and imputation can infer a more dense genotype, enabling broader uptake of genotyping technology by cattle producers [16,17]. The evolution of genotyping technology has resulted in many animals of different breeds being genotyped with a variety of SNP panels. For effective genomic selection, all animals should have genotypes of equivalent density. It has been shown that there is a need to evaluate different panels for imputation to higher density panels. Imputation also eliminates the need for re-genotyping of key animals, reducing costs of genomic selection and association analysis.

The Brazilian cattle industry plays a significant role in the national economy. Brazil has a herd of more than 211 million cattle of which 80% is zebu cattle [18]. Hereford and Braford breeds, together with Angus and Brangus account for 50% of the approximate 8 million doses of beef cattle semen commercialized in Brazil in 2013 [19]. Much of this semen, as well as most live bulls sold are mated to Zebu females with the primary objective of improving carcass quality [20].

The main objective of this research was to assess accuracy of imputation from lower density SNP panels to genotypes from the Illumina BovineSNP50 and the Illumina BovineHD panels (Illumina Inc., San Diego, USA) in Brazilian Braford and Hereford cattle.

## Methods

### Animal welfare

Animal welfare and use committee approval was not necessary for this study because data were obtained from existing databases.

### Data

Data was from the Conexão Delta G’s genetic improvement program - Hereford and Braford (Zebu x Hereford) cattle (Conexão Delta G, Dom Pedrito/RS, Brazil), containing approximately 520,000 animals from 97 farms located in the South, Southeast, Midwest and Northeast regions of Brazil. A total of 683 Hereford and 2,997 Braford animals from these farms were genotyped. Of the genotyped animals, there were 624 Hereford and 2,926 Braford animals genotyped with the Illumina BovineSNP50 panel, and 59 Hereford and 71 Braford animals genotyped with the Illumina BovineHD panel from 17 farms located in the South of Brazil. Data also included 88 Nellore bulls from the Paint Program (Lagoa da Serra, Sertãozinho/SP, Brazil) genotyped with the Illumina BovineHD panel.

### Data editing

For imputation to the 50K SNP panel, animals genotyped with 777K SNP genotypes had SNPs not contained on the 50K SNP panel removed. This resulted in a population of 3,768 animals genotyped for 49,345 SNPs. Sites were filtered for GenCall score (> = 0.15) [21,22], Call Rate (> = 0.90) [21,22] and Hardy-Weinberg Equilibrium (P > =10^−6^) [23,24]. Only autosomes were considered [3,4]. The individual sample quality control considered GenCall Score (> = 0.15) [21,22], Call Rate (> = 0.90) [21,22], heterozygosity deviation [21] (limit of ± 3 SD), repeated sampling and paternity errors [22]. After quality control, 3,698 animals and 43,248 SNP were used for further analysis.

For imputation to the 777K SNP panel, only the animals genotyped with the 777K SNP panel could be used as reference. The SNP quality control was the same as for the imputation to the 50K SNP panel (SNP in the 50K panel that were not in common with the 777K were also removed from 50K). After the quality control, 218 bulls (Hereford = 59, Braford = 71, Nellore = 88) and 587,620 SNPs remained.

Table 1 shows the numbers of genotyped animals after data editing as well as the pedigree structure of the genotyped animals.

**Table 1:** **Summary statistics of genotyped animals and pedigree structure of the 50K and the 777K SNP panels**

**Parameter**	**Braford**	**Hereford**	**Nellore**
*Imputation to the 50K SNP panel*			
Total of genotyped animals	2,946	664	88
Sires	39	29	6
Dams	76	21	0
Offspring	2,831	614	82
Offspring with sire and/or dam genotyped (%)	22.81	32.68	12.50
Average number of offspring per sire	15.28 ± 17.38	6.76 ± 6.46	1.83 ± 0.90
Smallest and largest number of offspring per sire	1-76	1-26	1-3
Average number of offspring per dam	1.00 ± 0.00	1.00 ± 0.00	1.00 ± 0.00
Offspring with sire and/or dam unknown (%)	69.86	48.04	18.18
*Imputation to the 777K SNP panel*			
Total of genotyped animals	71	59	88
Sires	8	3	5
Dams	0	0	0
Offspring	63	56	83
Offspring with sire and/or dam genotyped (%)	25.35	8.47	10.23
Average number of offspring per sire	2.25 ± 1.09	1.67 ± 0.94	1.80 ± 0.98
Smallest and largest number of offspring per sire	1-4	1-3	1-3
Average number of offspring per dam	0.00 ± 0.00	0.00 ± 0.00	0.00 ± 0.00
Offspring with sire and/or dam unknown (%)	53.52	38.98	18.18

### Reference and imputation populations

For imputation to the 50K SNP panel, the dataset was split into two populations. The imputation population was comprised of all animals born in 2011. The remainder of the population was assigned to the reference population for imputation. This division resulted in 2,735 animals in the reference population when Nellore animals were included and 2,647 when Nellore animals were not included. A total of 963 animals were sorted into the imputation population.

Hereford and Braford animals in the reference population included 129 sires born before 2008 and 2,518 animals born between 2008 and 2010. From these 2,518 animals, 3.8% had at least one genotyped offspring.

For animals in the imputation population, the 3K, 6K, 8K, 15K and 20K low density SNP panels were created by masking the non-overlapping SNP between the 50K SNP panel and each of these SNP panels. The imputation population included 33 animals with two parents genotyped and 308 animals with one parent genotyped. Moreover, 52% of the imputation animals were offspring of multiple sire matings.

The data set for imputation to the 777K SNP panel contained 71, 59 and 88 Braford, Hereford and Nellore animals, respectively. The strategy used to test the imputation was to create three different data sets randomly alternating animals in the reference population and in the imputation population, always keeping the Nellore animals in reference population as the objective was to test the imputation accuracy of Braford and Hereford cattle. Each reference population was composed by 175 animals (88 Nellore plus 87 Hereford and Braford animals) and each imputation population had 43 Hereford and Braford animals. For animals in the imputation population the 3K, 6K, 8K, 15K, 20K, 50K, 90iK and 90tK SNP panels were created by masking non-overlapping SNP from 777K SNP panel.

All panels, but one, were commercial panels: Illumina Bovine3K (3K), Illumina BovineLD (6K), Illumina BovineSNP50 (50K) and Illumina BovineHD (777K) panels (Illumina Inc., San Diego, USA), Beef LD GGP (8K), Indicus LD GGP (20K), GGP Taurus HD (90tK) and GGP Indicus HD (90iK) panels (Gene Seek Inc., Lincoln, USA) (Table 2).

**Table 2:** **Number of SNPs on each simulated panel before and after quality control for imputation to 50K or 777K SNP panels** ^**1**^

**Commercial name**	**Label**	**Number of SNPs**	**Number of SNPs in the imputation to 50K**	**Number of SNPs in the imputation to 777K**
Illumina Bovine3K	3K	2,900	2,321	2,359
Illumina BovineLD	6K	6,909	6,205	6,216
Beef LD GeneSeek Genomic Profiler	8K	8,762	7,033	7,478
15K panel^2^	15K	14,195	12,304	12,345
Indicus LD GeneSeek Genomic Profiler	20K	19,721	7,320	16,047
Illumina BovineSNP50	50K	54,609	43,247	43,247
GeneSeek Genomic Profiler Indicus HD	90iK	74,085	-	55,819
GeneSeek Genomic Profiler Beef HD	90tK	76,992	-	61,445
Illumina BovineHD	777K	787,799	-	587,620

^1^The SNP quality control included GenCall score (> = 0.15), Call Rate (> = 0.90), Hardy-Weinberg Equilibrium (P > =10^−6^), removal of non-autosomal chromosomes and SNPs not in common with reference panel;

^2^Non commercial panel. The 15K panel was created based on the Beef LD GeneSeek Genomic Profiler (8K) panel by expanding it with SNPs selected based on minor allele frequency greater than 0.23, linkage disequilibrium less than 0.088 and preferably located evenly spaced between two SNPs in the 8K SNP panel.

All the SNPs from 8K SNP panel were part of the customized 15K SNP panel. The remaining SNPs (7K) were selected from the 50K SNP panel using high minor allele frequency, low linkage disequilibrium, and location (approximately evenly spaced between two SNPs in the 8K SNP panel) as selection criteria. The best possible threshold values to meet the three criteria were a minor allele frequency greater than 0.23 and a linkage disequilibrium, as measured by r^2^, less than 0.088.

### Imputation scenarios

For imputation to the 50K SNP panel, four different scenarios were explored as follows: including Nellore genotypes in the reference population and either including pedigree information (NE-P) or not including pedigree information (NE-NP); not including Nellore genotypes in the reference population and either including pedigree information (NNE-P) or not including pedigree information (NNE-NP).

For imputation to the 777K SNP panel, a third set of Hereford and Braford bulls were imputed in four different scenarios: including Nellore genotypes and pedigree information in the reference population (NE-P) or including Nellore genotypes and not including pedigree information in the reference population (NE-NP). Each of these two scenarios was carried out in one or two steps. Two-step imputation was carried out only for panels with density less than 50K SNP. Two-step imputation involved: 1) in the first step, the animals genotyped with 3K, 6K, 8K, 15K and 20K SNP panels were imputed to the 50K SNP panel using in the reference population all the animals genotyped with the 50K SNP panel; 2) in the second step, all the animals imputed to the 50K SNP panel were then imputed to the 777K SNP panel using as reference two-thirds of the Hereford and Braford and all Nellore bulls genotyped with the 777K SNP panel. One-step imputation was performed by imputing from the simulated low density panels directly to the 777K SNP panel.

Imputation accuracy of above scenarios was assessed by concordance rate (CR), which corresponds to the proportion of genotypes correctly imputed, and by allelic R^2^, which corresponds to the square of the correlation between the number of minor alleles in the imputed genotype and the number of minor alleles in the original genotype [25].

There were thirty imputation scenarios from low density panels to the 50K SNP panel. Twenty-four scenarios were examined for imputation from low and medium density panels to 777K SNP panel and thirty scenarios were used to assess differences in imputation accuracy in one or two steps (Table 3).

**Table 3:** **Imputation scenarios used in the study**

**Imputation**		**Software**	**Pedigree information**	**Nellore genotypes**	**Method**
**From**	**To**				
3K, 6K, 8K,15K, 20K	50K	FImpute	Yes	Yes	One-step
				No	
			No	Yes	
				No	
		Beagle	No	Yes	
				No	
3K, 6K, 8K,15K, 20K	777K	FImpute	Yes	Yes	One-step
			No		
					Two-step
		Beagle	No		
50K, 90iK, 90tK	777K	FImpute	Yes	Yes	One-step
			No		
		Beagle	No		

### Imputation methods

Imputation was carried out by FImpute v.2.2 [11] and Beagle v.3.3 [8]. Beagle was used in scenarios that did not include pedigree information and ungenotyped animals. FImpute was used in all scenarios.

Imputation methods can be based on linkage disequilibrium information between markers in the population, but also can use the inheritance information within family. Beagle software is based on linkage disequilibrium between markers in the population and uses a Hidden Markov model [26] for inferring haplotype phase and filling in genotypes. Beagle also exploits family information indirectly by searching for long haplotypes. Contrary to Beagle, FImpute software uses a deterministic algorithm and makes use of both family and population information directly. Family information is taken into account only when pedigree information is available. The population imputation in FImpute is based on an overlapping sliding window method [11] in which information from close relatives (long haplotype match) is first utilized and information from more distant relatives is subsequently used by shortening the window size. The algorithm assumes that all animals are related to each other to some degree ranging from very close to very distant relationships.

### Comparison between scenarios

Analysis of variance was carried out using the GLM procedure in SAS version 9.2 (SAS Inst. Inc., Cary, NC) to compare the average CR and allelic R^2^ of each scenario. An arcsine square root [27] transformation was applied to CR and allelic R^2^ to normalize the residuals.

## Results

Of the 3,698 animals genotyped with the 50K SNP panel, ~24% had sire and/or dam genotyped and ~65% had at least one parent unknown in the pedigree. With respect to the animals genotyped with the 777K SNP panel, ~15% had sire and/or dam genotyped and ~35% had at least one parent unknown. Table 1 shows pedigree structure for each breed.

Table 4 provides the computing run time for each imputation scenario. Using FImpute, the run-time ranged between 2 and 48 minutes for different scenarios, while Beagle took between 25 and 2,280 minutes for the same scenarios. Table 5 provides the means and standard deviations of CR and allelic R^2^ for imputation to 50K and 777K SNP panels.

**Table 4:** **Overall computing run time in minutes for the different imputation scenarios** ^**1,2**^

**Panel**	**FImpute**				**Beagle**	
	**NE-P**	**NNE-P**	**NE-NP**	**NNE-NP**	**NE-NP**	**NNE-NP**
	*Imputation to the 50K SNP panel* ^3^					
3K	2	6	41	39	2280	2131
6K	3	7	46	45	828	772
8K	3	7	45	45	808	656
15K	3	9	48	48	328	317
20K	3	7	37	42	708	622
	*Imputation to the 777K SNP panel* ^4,5^					
3K	16 (17,24)	-	4 (5,8)	-	64 (224,41)	-
6K	17 (23,24)	-	4 (19,21)	-	49 (238,33)	-
8K	17 (23,24)	-	3 (20,23)	-	45 (177,34)	-
15K	15 (24,23)	-	8 (20,23)	-	40 (127,42)	-
20K	17 (23,23)	-	9 (20,23)	-	44 (161,42)	-
50K	3	-	11	-	29	-
90iK	17	-	11	-	25	-
90tK	17	-	10	-	33	-

^1^Run time based on 10 parallel jobs with computer with 4*6-core processors (Intel Xeon X5690 @ 3.47GHz) and 128 Gigabytes of memory in OS x86-64 GNU/Linux;

^2^Scenarios for imputation. (NE-P) - using Nellore genotypes in the reference population and considering pedigree information; (NNE-P) - not using Nellore genotypes in the reference population and considering pedigree information; (NE-NP) - using Nellore genotypes in the reference population and not using pedigree information; (NNE-NP) - not using Nellore genotypes in the reference population and not using pedigree information;

^3^2,735 or 2,647 (not using Nellore genotypes) animals in the reference population and 963 animals in the imputation population;

^4^Values outside the brackets refer to the one-step imputation. The reference and imputation population were formed by 175 and 43 animals, respectively;

^5^Values inside the brackets refer to the two-step imputation. The reference population were formed by 3,567 in the imputation from low density panel to the 50K SNP panel and 175 animals in the imputation from the 50K SNP panel to the 777K SNP panel. The imputation population was formed by 43 animals.

**Table 5:** **Mean and standard deviation (SD) of concordance rate and allelic R** ^**2**^ **calculated for different algorithms, panel densities and scenarios for both imputation to 50K and 777K SNP panels**

		**CR**		**Allelic R** ^**2**^	
	**No.**	**Mean**	**SD**	**Mean**	**SD**
		*Imputation to the 50K SNP panel*			
*Algorithm*					
Beagle	10	0.927	0.042	0.890	0.067
Fimpute	20	0.943	0.038	0.912	0.061
*Panel*					
3K	6	0.864	0.011	0.787	0.016
6K	6	0.946	0.008	0.919	0.011
8K	6	0.952	0.008	0.927	0.011
15K	6	0.973	0.006	0.962	0.008
20K	6	0.953	0.008	0.929	0.011
*Scenario*					
NE-P	5	0.943	0.041	0.913	0.065
NE-NP	10	0.935	0.041	0.901	0.066
NNE-P	5	0.943	0.042	0.912	0.067
NNE-NP	10	0.935	0.042	0.901	0.066
		*Imputation to the 777K SNP panel*			
*Algorithm*					
Beagle	8	0.895	0.040	0.826	0.066
Fimpute	16	0.921	0.035	0.866	0.059
*Panel*					
3K^1^	3	0.838	0.017	0.728	0.025
6K^1^	3	0.898	0.016	0.829	0.025
8K^1^	3	0.902	0.017	0.836	0.026
15K^1^	3	0.918	0.017	0.863	0.027
20K^1^	3	0.903	0.017	0.837	0.026
50K	3	0.930	0.016	0.882	0.025
90iK	3	0.952	0.010	0.919	0.016
90tK	3	0.955	0.009	0.925	0.014
*Scenario*					
NE-P	8	0.9199	0.037	0.865	0.062
NE-NP	16	0.9082	0.039	0.846	0.065
*Step*					
One-step	15	0.8064	0.884	0.674	0.147
Two-step	15	0.8920	0.032	0.819	0.053

^1^Means and standard deviation for the two-step analysis.

### Imputation of the low density panels to the 50K SNP panel

There were significant differences (P < 0.05) in CR and allelic R^2^ between the two algorithms and between pairs of simulated low density panels, as well as a significant algorithm by panel interaction (P < 0.05). However, there were no significant differences (P > 0.05) in CR and allelic R^2^ between scenarios (Table 6).

**Table 6:** **Analysis of variance performed on the average concordance rate and allelic R** ^**2**^ **of the animals in the imputation population from each scenario for imputation from low density panels to the 50K SNP panel** ^**1,2**^

**Concordance rate**			**Allelic R** ^**2**^		
**Source**	**Mean**	**Scheffé test** ^**3**^	**Source**	**Mean**	**Scheffé test** ^**3**^
*Algorithm* ^*4*^ *(P-value < 0.0001)*			*Algorithm* ^*4*^ *(P-value?<?0.0001)*		
FImpute	1.340	a	FImpute	1.283	a
Beagle	1.306	b	Beagle	1.244	b
*Panel* ^*5*^ *(P-value < 0.0001)*			*Panel* ^*5*^ *(P-value?<?0.0001)*		
15K	1.402	a	15K	1.368	a
20K	1.347	b	20K	1.295	b
8K	1.345	c	8K	1.292	c
6K	1.332	d	6K	1.276	d
3K	1.189	e	3K	1.085	e
*Scenario* ^*6*^ *(P-value 0.0147)*			*Scenario* ^*6*^ *(P-value 0.0277)*		
NE-P	1.323	a	NE-P	1.264	a
NNE-P	1.323	a	NE-NP	1.263	a
NE-NP	1.323	a	NNE-P	1.264	a
NNE-NP	1.322	a	NNE-NP	1.262	a
Algorithm*Panel (P-value < 0.0001)			Algorithm*Panel (P-value 0.0265)		
FImpute - 15K	1.420	a	FImpute -15K	1.388	a
Beagle - 15K	1.384	b	Beagle - 15K	1.347	b
FImpute - 20K	1.365	c	FImpute - 20K	1.316	c
FImpute - 8K	1.362	d	FImpute - 8K	1.312	d
FImpute - 6K	1.349	e	FImpute - 6K	1.295	e
Beagle - 20K	1.330	f	Beagle - 20K	1.275	f
Beagle - 8K	1.328	f	Beagle - 8K	1.272	f
Beagle - 6K	1.316	g	Beagle - 6K	1.257	g
FImpute - 3K	1.204	h	FImpute - 3K	1.104	h
Beagle - 3K	1.174	i	Beagle - 3K	1.067	i

^1^Concordance rate and allelic R^2^ were arcsine square root transformed for the analyses;

^2^Interactions between Algorithm*Scenario and Panel*Scenario were not statistically significant (P?>?0.05);

^3^Different letters within a group means that there is a statistical difference between two means (P?<?0.05);

^4^Algorithm used was either FImpute v.2.2 [11] or Beagle v.3.3 [8];

^5^3K, 6K, 8K, 15K and 20K are low-density panels;

^6^Scenarios for imputation to the 50K SNP panel. (NE-P) - using Nellore genotypes in the reference population and considering pedigree information; (NNE-P) - not using Nellore genotypes in the reference population and considering pedigree information; (NE-NP) - using Nellore genotypes in the reference population and not using pedigree information; (NNE-NP) - not using Nellore genotypes in the reference population and not using pedigree information.

The non-commercial 15K SNP panel resulted in the highest imputation accuracy of the low density panels with an overall CR of 0.973 and allelic R^2^ of 0.962, 0.109 and 0.175 points higher than the 3K SNP panel, respectively (Table 5). The use of Nellore genotypes or use of pedigrees in FImpute did not improve CR or allelic R^2^ when imputing to the 50K SNP panel (Table 6). The average CR and allelic R^2^ for the four scenarios were 0.940 and 0.905, respectively. Using FImpute resulted in an overall average CR of 0.943 and allelic R^2^ of 0.912 while for Beagle the same average features were 0.927 and 0.890, respectively (Table 5). The algorithm by panel interaction, showed larger differences in CR and allelic R^2^ between FImpute and Beagle for sparser panels (0.021 in CR and 0.031 in allelic R^2^ for the 3K SNP panel) when compared to denser panels (0.012 in CR and 0.016 in allelic R^2^ for the 15K SNP panel), with FImpute being consistently more accurate. Imputation accuracy for 8K and 20K SNP panels were not significantly different using Beagle (P > 0.05) with respect to CR and allelic R^2^ (Table 6). The highest CR (>0.977) and allelic R^2^ (>0.967) were obtained using the 15K SNP panel and FImpute.

**Figure 1:**
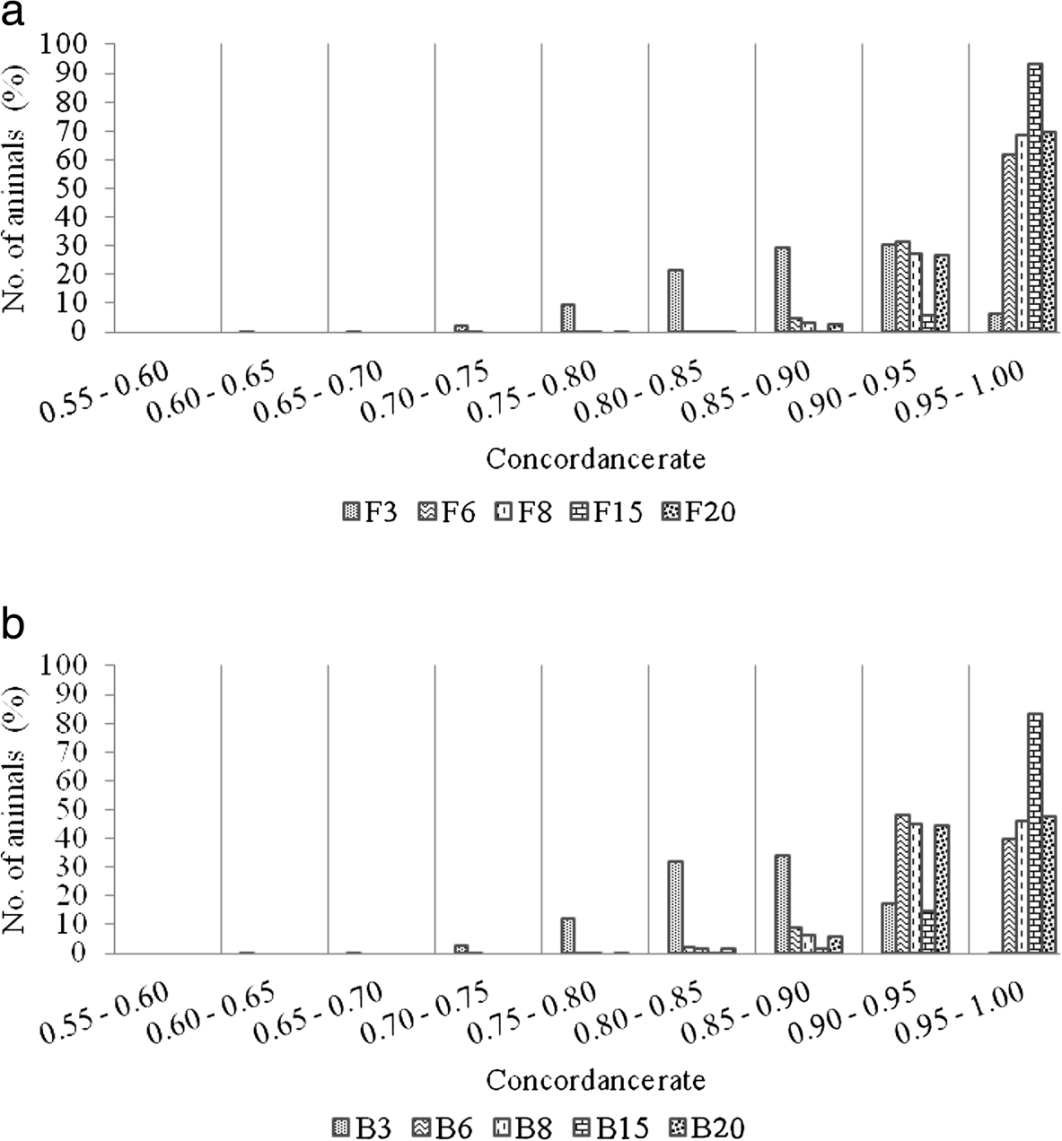
**Concordance rate of imputation to the 50K panel in different concordance rate bins.** Average over scenarios of imputation from alternative low density panels (3K, 6K, 8K, 15K and 20K) to the 50K SNP panel. **a)** using FImpute; **b)** using Beagle.

An important measurement of imputation success is the number of animals imputed with modest accuracy (assumed <0.950 CR here). Using the 15K SNP panel resulted in 93% and 83% of the animals being imputed with a CR above 0.950 (average of all scenarios) for FImpute and Beagle, respectively, while using the 3K SNP panel as the low density panel resulted in only 6.3% and 0.8% of animals above this accuracy threshold using FImpute and Beagle, respectively. The results for the other panels ranged between 62% and 70% using FImpute and between 40% and 48% using Beagle (Figure 1).

**Figure 2:**
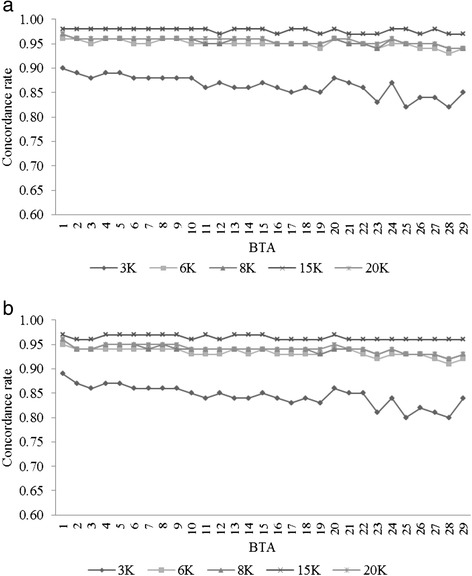
**Concordance rate of imputation to the 50K panel for all BTAs and scenarios. a)** using FImpute; **b)** using Beagle.

The CR (average of all scenarios) for the 3K SNP panel, from either FImpute or Beagle, were lower than all other panels with CR values over all BTAs at or below 0.900. All other panels produced CR above 0.930 for all chromosomes. Imputation accuracy was found to be relative to chromosome length with the highest CRs obtained for BTA1 while the lowest CRs were obtained for BTA28 in all scenarios and both algorithms, however little difference was seen across the genome (Figure 2).

**Figure 3:**
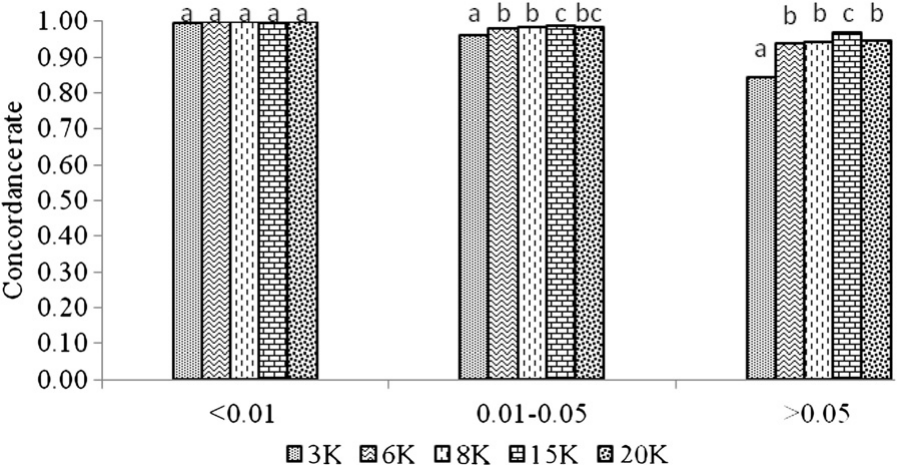
**Concordance rate of imputation by MAF classes.** Average over scenarios of imputation from alternative low density panels (3K, 6K, 8K, 15K and 20K) to the 50K SNP panel. Within a group of colums, two different letters means a statistical difference (P < 0.05).

The average CR for imputation from the alternative low density panels (3K, 6K, 8K, 15K and 20K) to the 50K SNP panel was calculated for three different classes of minor allele frequency (MAF) (<0.01, 0.01-0.05, and >0.05). For the MAF class <0.01 the average CR was close to 1.00 for all panel densities. For SNPs with MAF 0.01-0.05 and >0.05 the average CRs ranged similarly from 0.84 to 0.97, depending on the panel density (Figure 3).

### Imputation of the ungenotyped animals to the 50K SNP panel

FImpute allows for accurate imputation of 50K genotypes for ungenotyped animals that have four or more offspring [11]. Thirty-seven animals that had four or more offspring were imputed and showed an average CR of 0.950 and with 99.86% of the SNPs imputed. When average CR were examined based on the number of offspring, accuracies of 0.924, 0.941, 0.972, 0.961 and 0.990 were found for bulls with 4–9, 10–19, 20–29, 30–39 and over 40 offspring, respectively. There were 11, 11, 9, 3 and 3 bulls in each of those progeny size classes, respectively. The lowest CR (0.900) corresponded to two Hereford animals with five offspring each, while the highest CR (above 0.980) was for six Braford animals with more than twenty offspring each.

### Imputation of the low density panels to the 777K SNP panel

There were significant differences (P < 0.05) in CR and allelic R^2^ between algorithms, panels and scenarios when imputing to 777K SNP panel. The algorithm by panel interaction was also significant (P < 0.05) (Table 7).

**Table 7:** **Analysis of variance performed on the average concordance rate and allelic R** ^**2**^ **of the animals in the imputation population from each scenario for imputation from low density panels to the 777K SNP panel** ^**1,2,3**^

**Concordance rate**			**Allelic R** ^**2**^		
**Source**	**Mean**	**Scheffé test** ^**4**^	**Source**	**Mean**	**Scheffé test** ^**4**^
*Algorithm* ^*5*^ *(P-value < 0.0001)*			*Algorithm* ^*5*^ *(P-value?<?0.0001)*		
FImpute	1.291	a	FImpute	1.203	a
Beagle	1.244	b	Beagle	1.145	b
*Panel* ^*6*^ *(P-value < 0.0001)*			*Panel* ^*6*^ *(P-value?<?0.0001)*		
90tK	1.351	a	90tK	1.286	a
90iK	1.343	b	90iK	1.275	b
50K	1.295	c	50K	1.210	c
15K	1.273	d	15K	1.181	d
20K	1.247	e	20K	1.146	e
8K	1.245	e	8K	1.144	e
6K	1.239	e	6K	1.135	e
3K	1.150	f	3K	1.013	f
*Scenario* ^*7*^ *(P-value 0.0258)*			*Scenario (P-value 0.0346)*		
NE-NP	1.269	a	NE-NP	1.175	a
NE-P	1.267	b	NE-P	1.172	b
Algorithm*panel (P-value =0.0052)			Algorithm*panel (P-value =0.0107)		
FImpute - 90tK	1.370	a	FImpute - 90tK	1.309	a
FImpute - 90iK	1.364	a	FImpute - 90iK	1.301	a
Beagle - 90tK	1.331	b	Beagle - 90tK	1.262	b
FImpute - 50K	1.322	b	Beagle - 90iK	1.249	b
Beagle - 90iK	1.322	b	FImpute - 50K	1.244	b
FImpute - 15K	1.300	c	FImpute - 15K	1.215	c
FImpute - 20K	1.271	d	Beagle - 50K	1.176	d
FImpute - 8K	1.269	d	FImpute - 20K	1.176	d
Beagle - 50K	1.269	d	FImpute - 8K	1.174	d
FImpute - 6K	1.262	d	FImpute - 6K	1.165	d
Beagle - 15K	1.245	e	Beagle - 15K	1.146	e
Beagle - 20K	1.222	f	Beagle - 20K	1.115	f
Beagle - 8K	1.221	f	Beagle - 8K	1.114	f
Beagle - 6K	1.215	f	Beagle - 6K	1.106	f
FImpute - 3K	1.169	g	FImpute - 3K	1.039	g
Beagle - 3K	1.130	h	Beagle - 3K	0.988	h

^1^Concordance rate and allelic R^2^ were arcsine square root transformed for the analyses;

^2^Interaction effects between Algorithm*Scenario and Panel*Scenario were not statistically significant (P?>?0.05);

^3^3K, 6K, 8K, 15K and 20K are low-density panels were imputed in two steps (firstly they were imputed to the 50K and then to the 777K SNP panel);

^4^Different letters within a group means that there is a statistical difference between two means (P?<?0.05);

^5^Algorithm used was either FImpute v.2.2 [11] or Beagle v.3.3 [8];

^6^3K, 6K, 8K, 15K, 20K, 50K, 90iK and 90tK are low-density panels;

^7^Scenarios for imputation to the 777K SNP panel. (NE-P) - using Nellore genotypes in the reference population and considering pedigree information; (NE-NP) - using Nellore genotypes in the reference population and not using pedigree information.

Using FImpute resulted in an overall average CR of 0.921 and allelic R^2^ of 0.866, while Beagle yielded an average CR of 0.895 and allelic R^2^ of 0.826 (Table 5). The 6K, 8K and 20K SNP panels did not significantly differ (P > 0.05) in their average CR and allelic R^2^ (Table 7). The highest CR and allelic R^2^ were obtained with the 90tK SNP panel (CR = 0.955; allelic R^2^ = 0.925) and the lowest CR and allelic R^2^ with the 3K SNP panel (CR = 0.838; allelic R^2^ = 0.728). For the other panels, CR was between 0.898 and 0.952 and allelic R^2^ was between 0.829 and 0.919 (Table 5). The use of the pedigree information (NE-P) slightly decreased the CR and allelic R^2^ for imputation to the 777K SNP panel (P < 0.05) (Table 7). The interaction algorithm by panel, showed larger differences in CR and allelic R^2^ between FImpute and Beagle for sparse panels (0.028 in CR and 0.044 in allelic R^2^ for the 3K SNP panel) when compared to denser panels (0.016 in CR and 0.024 in allelic R^2^ for the 90tK SNP panel), with FImpute resulting in consistently higher accuracy of imputation.

**Figure 4:**
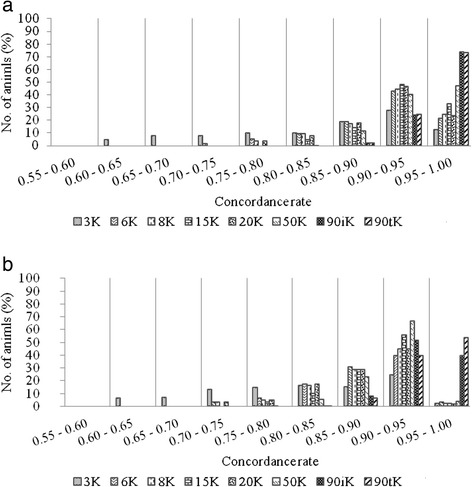
**Concordance rate of imputation to the 777K panel in different concordance rate bins.** Average over scenarios of imputation from alternative low density panels (3K, 6K, 8K, 15K, 20K, 50K, 90iK and 90tK) to the 777K SNP panel. **a)** using FImpute; Please note that figures cannot be composed of text only. Since it is in a table format, please modify Figure 1 as a normal table with at least two columns. Please ensure that if there are other tables in the manuscript, affected tables and citations should be renumbered in ascending numerical order. using Beagle.

The distributions of animals in high classes of CR varied between FImpute and Beagle. For FImpute, the proportion of animals imputed above a CR of 0.95 ranged from 12.8% for the 3K SNP panel to 73.6% for the 90iK SNP panel. For the other panels, the proportion of animals was between 20% and 48% (Figure 4a). For Beagle, with the exception of the 90iK SNP panel (39.5%) and the 90tK SNP panel (53.5%), the proportion of animals imputed above a CR of 0.95 was around 3% (Figure 4b).

**Figure 5:**
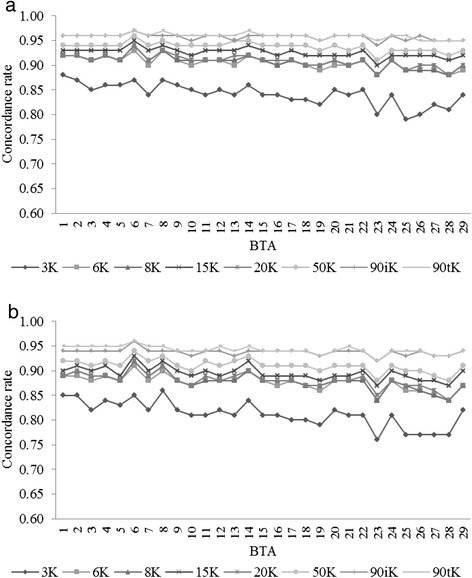
**Concordance rate of imputation to the 777K panel for all BTAs and scenarios. a)** using FImpute; **b)** using Beagle.

Imputation accuracy per chromosome using Beagle was only greater than 0.900 when 50K or more dense panels were used (Figure 5b), while the same was observed using FImpute for all panels denser than 6K (Figure 5a). Per chromosome accuracies followed the results from 50K, where the highest accuracy was observed on BTA1, and the lowest on BTA28.

Imputation to the 777K SNP panel performed in two steps was statistically superior (P < 0.05) to imputation in a one-step both when measured by CR and allelic R^2^, and this difference was observed for all scenarios (Table 8). The interaction between number of steps and algorithm showed larger difference between CR and allelic R^2^ from one and two steps imputation when Beagle was used (0.107 in CR and 0.181 in allelic R^2^). The interaction between number of steps and low density panel showed that the difference between CR and allelic R^2^ from one to two steps imputation was larger for sparse panels (0.178 in CR and 0.298 in allelic R^2^ for the 3K SNP panel) when compared to denser panels (0.020 in CR and 0.034 in allelic R^2^ for the 20K SNP panel.

**Table 8:** **Analysis of variance performed on the average concordance rate and allelic R** ^**2**^ **of the animals in the imputation population from each scenario for imputation to the 777K SNP panel by one or two steps** ^**1,2**^

**Concordance rate**			**Allelic R** ^**2**^		
**Source**	**Mean**	**Scheffé test** ^**3**^	**Source**	**Mean**	**Scheffé test** ^**3**^
*Step* ^*4*^ *(P-value < 0.0001)*			*Step* ^*4*^ *(P-value?<?0.0001)*		
Two-step	1.231	a	Two-step	1.125	a
One-step	1.110	b	One-step	0.997	b
*Algorithm* ^*5*^ *(P-value < 0.0001)*			*Algorithm* ^*4*^ *(P-valeu 0.0001)*		
FImpute	1.202	a	FImpute	1.080	a
Beagle	1.140	b	Beagle	0.997	b
*Panel* ^*6*^ *(P-value < 0.0001)*			*Panel* ^*6*^ *(P-value?<?0.0001)*		
15K	1.236	a	15K	1.130	a
20K	1.229	b	20K	1.120	a
8K	1.180	c	8K	1.052	b
6K	1.167	d	6K	1.034	c
3K	1.042	e	3K	0.855	d
*Scenario* ^*7*^ *(P-value 0.7638)*			*Scenario* ^*7*^ *(P-value 0.9983)*		
NE-NP	1.171	a	NE-NP	1.038	a
NE-P	1.170	a	NE-P	1.038	a
Step*Algorithm (P-value < 0.0001)			Step*Algorithm (P-value?<?0.0001)		
Two-step - FImpute	1.254	a	Two-step - FImpute	1.154	a
Two-step - Beagle	1.208	b	Two-step - Beagle	1.095	b
One-step - FImpute	1.149	c	One-step - FImpute	1.006	c
One-step - Beagle	1.072	d	One-step - Beagle	0.898	d
Step*Panel (P-value < 0.0001)			Step*Panel (P-value?<?0.0001)		
Two-step - 15K	1.274	a	Two-step - 15K	1.183	a
Two-step - 20K	1.247	b	Two-step - 20K	1.147	b
Two-step - 8K	1.246	b	Two-step - 8K	1.145	b
Two-step - 6K	1.239	b	Two-step - 6K	1.136	b
One-step - 20K	1.210	c	One-step - 20K	1.094	c
One-step - 15K	1.198	c	One-step - 15K	1.078	c
Two-step - 3K	1.149	d	Two-step - 3K	1.013	d
One-step - 8K	1.114	e	One-step - 8K	0.960	e
One-step - 6K	1.094	f	One-step - 6K	0.932	e
One-step - 3K	0.936	g	One-step - 3K	0.696	f

^1^Concordance rate and allelic R^2^ were arcsine square root transformed for the analyses;

^2^Interaction effects between step*scenario, algorithm*panel, algorithm*scenario and panel*scenario were not statistically significant (P?>?0.05);

^3^Different letters within a group means that there is a statistical difference between two means (P?<?0.05);

^4^One-step is the imputation from the low-density panels to the 777K SNP panel and two-step is the imputation from low-density panels to 50K SNP panel and after the imputation from 50K SNP panel to 777K SNP panel;

^5^Algorithm used was either FImpute v.2.2 [11] or Beagle v.3.3 [8];

^6^3K, 6K, 8K, 15K, and 20K are low-density panels;

^7^Scenarios for imputation to the 777K SNP panel. (NE-P) - using Nellore genotypes in the reference population and considering pedigree information; (NE-NP) - using Nellore genotypes in the reference population and not using pedigree information.

The relative increase in CR for the two-step imputation with respect to the one-step imputation was 27%, 12%, 11%, 5% and 2% for 3K, 6K, 8K, 15K and 20K SNP panels, respectively, and the relative increase in allelic R^2^ was 69%, 21% 22% 9% and 4% for 3K, 6K, 8K, 15K and 20K SNP panels, respectively.

**Figure 6:**
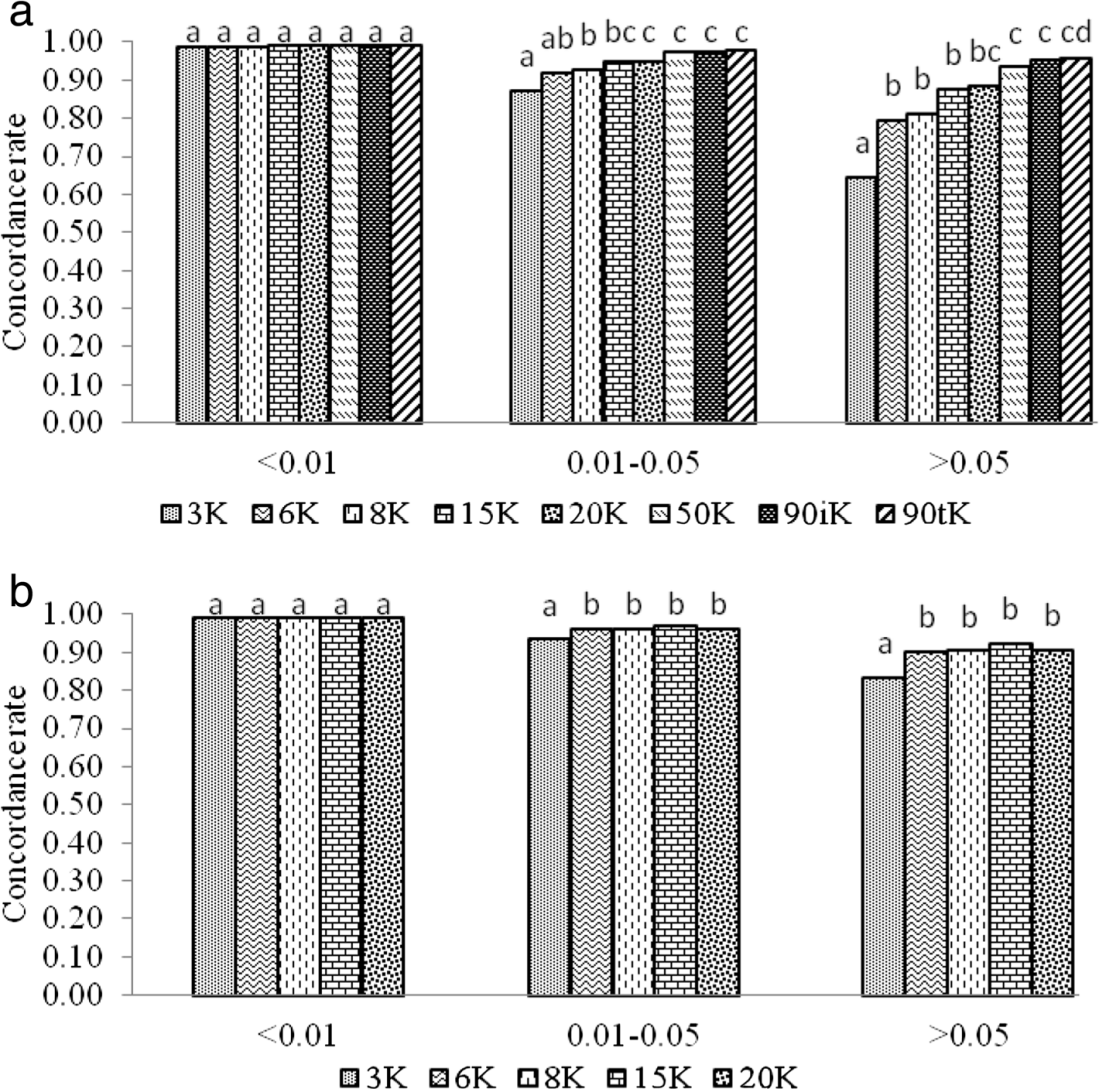
**Concordance rate of imputation by MAF classes. a)** Average over scenarios of imputation from alternative low density panels (3K, 6K, 8K, 15K and 20K, 50K, 90iK and 90tK) to the 777K SNP panel; **b)** Average over scenarios of imputation from alternative low density panels (3K, 6K, 8K, 15K, 20K) to the 777K SNP panel in two-step imputation. Within a group of colums, two different letters means a statistical difference (P < 0.05).

The average CR for imputation from the alternative low density panels (3K, 6K, 8K, 15K, 20K, 50K, 90iK and 90tK) to the 777K SNP panel was calculated for three different classes of MAF (<0.01, 0.01-0.05, and >0.05). For the MAF class <0.01 the average CR was close to 0.99 for all panel densities, for MAF class 0.01-0.05 and >0.05 the average CRs ranged from 0.84 to 0.97 and from 0.65 to 0.96, respectively, depending on the panel density (Figure 6).

## Discussion

### Imputation of the low density panels to the 50K SNP panel

There was no significant difference when imputation was performed using Nellore genotypes in the reference population and when the imputation was based on either family and population imputation or population imputation only. This means including pedigree information did not improve the CR and allelic R^2^ and is not required for accurate imputation. When Nellore genotypes were included in the reference population, it was expected that it would increase CR and allelic R^2^ because imputation population was mostly formed by Braford animals that have in their breed composition from 15% to 75% of zebu breeds, including the Nellore breed. This implies that the haplotypes present in the Braford animals available in the reference population are able to account for almost all of the haplotypes in the population. Ventura et al. [5] also did not find differences in imputation accuracies when the reference population included Angus plus multiple breeds or Charolais plus multiple breeds to impute crossbreds in Canada. Berry et al. [28], studying seven dairy and beef breeds in Ireland, concluded that reference populations formed by multiple breeds did not significantly increase the accuracy of the imputation of purebreds.

Including pedigree information did not increase CR or allelic R^2^. This could be expected due to the weak structure of the pedigree within the set of genotyped animals and in the whole pedigree file. Similar results were found by Carvalheiro et al. [21] when working with Nellore in Brazil with similar pedigree structure. However, Ma et al. [29] found increases in CR between 1% and 2% using Beagle and FImpute in Nordic Red cattle in Sweden when including genotypes of the bull-sires of the imputation population into the reference population. It would not, however, require pedigree information to detect these relationships in either algorithm.

The interaction between algorithm and panel was significant and yielded greater differences in CR and allelic R^2^ between FImpute and Beagle for low density panels, showing a greater advantage to using FImpute when a sparser low density panel is used. Carvalheiro et al. [21], working with Nellore in Brazil, also reported that FImpute outperformed Beagle for different low density panels and that there was a trend of greater differences between algorithms as low density panel density decreased.

The CR and allelic R^2^ values from FImpute in all analyses were consistently higher than those from Beagle, showing that the overlapping windows approach used by FImpute better infer missing genotypes than Hidden Markov models used by Beagle. Similar results were obtained by Carvalheiro et al. [21] in Nellore in Brazil and Larmer et al. [30], who worked on imputation from 6K and 50K SNP panels to 777K SNP panel in dairy cattle in Canada.

The 20K SNP panel was mainly developed for imputation to the 777K SNP panel and it has only 7,320 common SNPs with the 50K SNP panel. No difference between the 8K and 20K SNP panel was found using Beagle algorithm as they had similar number and average distance between the SNPs present on the 50K SNP panel. A few studies have tested the accuracy of imputation using different densities of markers and denser low density panels have consistently led to higher imputation accuracy in several beef cattle breeds, observed in Wang et al. [31] in Angus, Dassonneville et al. [17] in Blonde d’Aquitaine, Huang et al. [32] in Hereford and Chud [33] in Canchim cattle. The customized 15K SNP panel created in this study showed higher CR and allelic R^2^ when compared to the other low density panels, including the 20K SNP panel. The reason for that may because of a higher density of markers in low linkage disequilibrium with adjacent SNPs and medium to high minor allele frequency in the population, allowing a better haplotype reconstruction. The superiority of the customized 15K SNP panel in relation to the commercial panels, however, might be expected because it was created based on criteria specific for this population. Carvalheiro et al. [21], working with Nellore cattle in Brazil, also developed a 15K SNP panel for imputation to the 777K SNP panel. They found slightly better results when compared to imputation from the 50K SNP panel. One possible disadvantage of customized panels is the cost will likely be higher in comparison to already available commercial panels of similar density.

The highest accuracies were obtained for all low density panels when examining BTA1, whereas the worst results were obtained for BTA28. Sun et al. [34], working with Angus genotypes in the United States, reported that genotype imputation was more difficult in the initial and end regions of the chromosomes. Therefore, the shorter are the chromosomes, which is the case of BTA28 (46 Mb), the lower the overall chromosome accuracy, as the poorly imputed distal regions comprise a greater proportion of the overall chromosome. Similar results were found by Berry & Kearney [35] in Irish Holstein cattle, when imputing from the 3K to the 50K SNP panel. Moreover, Pausch et al. [24], working with Fleckvieh in Germany and imputing from 50K to 777K SNP panel, and Wang et al. [31], working with Angus in the United States and imputing to the 50K SNP panel from various low density panels, found higher and lower accuracies for BTA1 and BTA28, respectively, when compared to the average accuracy of imputation for all chromosomes.

### Imputation of low density panels to the 777K SNP panel

On average, the imputation population had seven animals with one of the parents genotyped and the reference population had twenty-four animals with one of the parents also genotyped. The inclusion of pedigree information did not result in an increase in CR and allelic R^2^. Carvalheiro et al. [21] studying, among other factors, the effect of using or not the pedigree information in Nellore, also did not observe significant difference in CR when imputing from 15K and 50K to the 777K SNP panel using FImpute.

The two-step imputation procedure consistently out-performed imputation in one-step. This result confirmed that more SNPs contained on the low density panel, results in greater accuracy of imputation [7,17,31,33]. Similar results were found by Larmer et al. [30] in Canadian Holstein cattle, when imputing in two steps from 6K to the 50K and from 50K to the 777K SNP panel. The interaction between algorithm and one or two steps was significant and showed greater difference in CR and allelic R^2^ between one and two steps methods when using Beagle. The percentage of animals with CR above 0.95, in general, was higher for higher density panels, as expected. However, the 15K SNP panel showed higher percentage than the 20K SNP panel, most likely due to the criteria that were used for developing the 15K SNP panel. Moreover, it may be also due to the fact that the 20K SNP panel was developed mainly for genotype imputation in *Bos Taurus Indicus* cattle.

The results by chromosome followed the same pattern found for imputation to the 50K SNP panel, with longer chromosomes having greater imputation accuracies [33,34].

### Imputation of the ungenotyped animals to the 50K SNP panel

Genotype imputation for ungenotyped animals is now a lower cost alternative that can be used to increase the training population towards the implementation of genomic selection. Important ungenotyped ancestors that may have no available biological material to perform genotyping can also be accurately imputed using genotyped progeny information. Also, groups of cows that were ungenotyped due to the costs can have their genotypes inferred [36-38]. Different software, such as AlphaImpute [39], FindHap [12], PedImpute [23] and FImpute [11] are able to infer genotypes ungenotyped animals with high CR using different approaches, such as imputation based on: genotyped parents; sire and maternal grandsire, dam and paternal grand dam, sire only, dam only, and offspring. However, the accuracy of each approach is different [36-38].

Ungenotyped animals in this study were imputed using FImpute, using offspring. FImpute requires at least 4 offspring be available for imputation of ungenotyped individuals (default parameter). Preliminary results obtained by Sargolzaei et al. [11] and Berry et al. [38] using FImpute clearly showed an inability to impute the genotype of sires when a paternal halfsib family size of three or less was used. However, the results indicated that the greater the number of genotyped offspring, the higher were the CR values.

These results were similar to the ones reported by Berry et al. [38] studying seven dairy and beef breeds in Ireland with five offspring per ungenotyped individual and Bouwman et al. [36] studying dairy cattle in Netherlands with four offspring per ungenotyped animal. The average value found in this study was compatible to what is considered an accurate imputation from low density, i.e. average CR above 0.950 and having a very low missing rate.

## Conclusions

All low density panels, except the 3K SNP panel, can be used to impute to the 50K SNP panel with average concordance rates higher than 0.940. The customized 15K SNP panel yielded the highest percentage of animals with concordance rate above 0.950 of all the low density panels studied.

The 50K, 90iK and 90tK SNP panels can be used to impute to the 777K SNP panel with average concordance rates higher than 0.940. A two-step imputation is recommended for lower density panels, making use of all available intermediate density panel genotypes.

FImpute outperformed Beagle in all scenarios for imputation to both the 50K and to the 777K SNP panels both in terms of accuracy and computing time required.

Ungenotyped animals that have four or more offspring and do not have available biological material to carry out genotyping may have their 50K SNP panel genotype inferred with an average concordance rate of 0.950 in the Hereford/Braford population analyzed.

## Abbreviations

BTA: Bos taurus autosomal chromosome
CR: Concordance rate
DNA: Deoxyribonucleic acid
GLM: General linear models
GGP: GeneSeek Genomic Profiler
K: Kbytes
MAF: Minor allele frequency
Mb: Mega base pairs
NC: North Carolina
SAS: Statistical analysis system
SD: Standard deviation
SNP: Single nucleotide polymorphism
USA: United States of America
